# Ethanol Inhibits High-Affinity Immunoglobulin E Receptor (FcεRI) Signaling in Mast Cells by Suppressing the Function of FcεRI-Cholesterol Signalosome

**DOI:** 10.1371/journal.pone.0144596

**Published:** 2015-12-14

**Authors:** Lubica Draberova, Tomas Paulenda, Ivana Halova, Lucie Potuckova, Viktor Bugajev, Monika Bambouskova, Magda Tumova, Petr Draber

**Affiliations:** Laboratory of Signal Transduction, Institute of Molecular Genetics, Academy of Sciences of the Czech Republic, Prague, Czech Republic; Cornell University, UNITED STATES

## Abstract

Ethanol has multiple effects on biochemical events in a variety of cell types, including the high-affinity immunoglobulin E receptor (FcεRI) signaling in antigen-activated mast cells. However, the underlying molecular mechanism remains unknown. To get better understanding of the effect of ethanol on FcεRI-mediated signaling we examined the effect of short-term treatment with non-toxic concentrations of ethanol on FcεRI signaling events in mouse bone marrow-derived mast cells. We found that 15 min exposure to ethanol inhibited antigen-induced degranulation, calcium mobilization, expression of proinflammatory cytokine genes (tumor necrosis factor-α, interleukin-6, and interleukin-13), and formation of reactive oxygen species in a dose-dependent manner. Removal of cellular cholesterol with methyl-β-cyclodextrin had a similar effect and potentiated some of the inhibitory effects of ethanol. In contrast, exposure of the cells to cholesterol-saturated methyl-β-cyclodextrin abolished in part the inhibitory effect of ethanol on calcium response and production of reactive oxygen species, supporting lipid-centric theories of ethanol action on the earliest stages of mast cell signaling. Further studies showed that exposure to ethanol and/or removal of cholesterol inhibited early FcεRI activation events, including tyrosine phosphorylation of the FcεRI β and γ subunits, SYK kinases, LAT adaptor protein, phospholipase Cγ, STAT5, and AKT and internalization of aggregated FcεRI. Interestingly, ethanol alone, and particularly in combination with methyl-β-cyclodextrin, enhanced phosphorylation of negative regulatory tyrosine 507 of LYN kinase. Finally, we found that ethanol reduced passive cutaneous anaphylactic reaction in mice, suggesting that ethanol also inhibits FcεRI signaling under *in vivo* conditions. The combined data indicate that ethanol interferes with early antigen-induced signaling events in mast cells by suppressing the function of FcεRI-cholesterol signalosomes at the plasma membrane.

## Introduction

Although it is known that ethanol has multiple effects on a variety of cells types, the molecular mechanisms of its action are far from understood. There are two basic theories of ethanol action on the cells, lipid-centric and protein-centric [1]. The lipid theory of ethanol action postulates that ethanol, similarly to anesthetics [2,3], dissolves in cellular lipids and acts by nonspecific mechanisms. This theory was supported by experiments showing that alcohols and anesthetics induce changes in properties of cellular membranes, including fluidity [4], lateral mobility of lipid molecules [5], phase transition temperature [6,7], and membrane permeability [8]. The protein theory of alcohol and anesthetics action proposes that the drugs interact specifically with certain proteins and in this way affect their properties [9]. This theory was mostly based on experiments suggesting that binding of alcohols and anesthetics induces conformational changes that diminish or abolish the function of some proteins, such as those forming neurotransmitter-gated ion channels [10–13]. However, concentrations of ethanol required to cause significant changes in the receptor functions were often greater than those attainable *in vivo*, and effects mediated by lower concentrations have not always been replicated [14].

Previous studies showed that alcohol modulates various components of the immune system [15–17]. Acute exposure to ethanol resulted in reduced monocyte/macrophage phagocytosis [18,19], T-cell receptor signaling [20], Toll-like receptor-mediated activation of macrophages [21,22], and reduced neutrophil migration [23]. It has also been reported that *in vitro* exposure of mast cells to ethanol for 1 hour or longer inhibited the high-affinity immunoglobulin E (IgE) receptor (FcεRI)-induced degranulation and production of tumor necrosis factor (TNF)-α and interleukin (IL)-8 [24,25]. Although these data suggested that ethanol inhibits signal transduction from the immunoreceptors, molecular mechanisms of the inhibitory action of ethanol on early steps of immunoreceptor signaling remained enigmatic.

In this study we used primary mouse bone marrow-derived mast cells (BMMCs) and examined sensitivity to ethanol of the earliest signaling events after FcεRI triggering. We also examined effect of ethanol on FcεRI activation in cells with reduced levels of cholesterol and on passive cutaneous anaphylaxis (PCA) in mice. Our data indicate that ethanol inhibits tyrosine phosphorylation of the FcεRI β and γ subunits, the first biochemically defined event after antigen-mediated aggregation of FcεRI, and support lipid-centric theory of ethanol action in mast cells.

## Materials and Methods

### Mice and cells

Mice were bred and maintained in specific pathogen free facility of the Institute of Molecular Genetics and used in compliance with the Institute guidelines. All protocols, including killing mice by decapitation, was approved by the Animal Care and Use Committee of the Institute of Molecular Genetics (Permit number 12135/2010-17210) and was in compliance with the EU Directive 2010/63/EU for animal experiments. All efforts were made to minimize suffering of the mice.

Bone marrow mast cells were isolated from femurs and tibias of C57BL/6 mice (females, 6–8 weeks old). The cells were cultured in RPMI-1640 medium supplemented with 100 U/ml penicillin, 100 μg/ml streptomycin, 71 μM 2-mercaptoethanol, minimum essential medium non-essential amino acids, 0.7 mM sodium pyruvate, 2.5 mM L-glutamine, 12 mM D-glucose, recombinant mouse stem cell factor (SCF; 20 ng/ml, ProSpec), mouse recombinant IL-3 (20 ng/ml, ProSpec) and 10% fetal calf serum (FCS). For PCA experiments, BALB/c male mice aged 8–12 weeks were used.

### Antibodies and reagents

The following monoclonal antibodies (mAbs) were used: anti-FcεRI β chain [26], trinitrophenol (TNP)-specific IgE mAb (IGEL b4 1) [27], anti-LAT [28], anti-LYN [29], and anti-NTAL [30]. Polyclonal antibodies specific for LYN and LAT were prepared by immunization of rabbits with the corresponding recombinant proteins or their fragments [31]. Rabbit anti-IgE was prepared by immunization with whole IGEL b4.1. Polyclonal antibodies specific for phospholipase C (PLC)γ1, phospho(p)PLCγ1 (Tyr 783), STAT5, ERK, pERK (Tyr 783), AKT, pAKT (Ser 473) as well as horseradish peroxidase (HRP)-conjugated goat anti-mouse IgG, and goat anti-rabbit IgG, were obtained from Santa Cruz Biotechnology Inc. Antibodies specific for pSYK (Tyr 525/Tyr 526), pSTAT5 (Tyr 694), pLYN (Tyr 507), and pLYN (Tyr 416) were obtained from Cell Signaling. Antibody specific for pLAT (Tyr 191) cross-reacting with pNTAL was obtained from Merc-Millipore. PAG-specific rabbit polyclonal antibody was from Exbio. V450-conjugated rat anti-mouse CD107a (LAMP1) and HRP-conjugated anti- phosphotyrosine mAb (PY-20) were obtained from BD Biosciences. Anti-mouse FcεRI labeled with fluorescein isothiocyanate (FITC) and anti-mouse KIT-allophycocyanin conjugates were obtained from eBiosciences. Donkey anti-mouse Ig-Alexa Fluor 568 conjugate was obtained from Invitrogen. All other reagents were from Sigma-Aldrich.

### Flow cytometry

For flow cytometry measurements, BMMCs (10^5^/sample) were used. To analyze the surface levels of FcεRI and Kit receptor, the labeling proceeded as described previously [32]. For analysis of surface CD107a (LAMP1), BMMCs were activated with various concentrations of antigen for 10 min at 37°C. Activation was stopped by pelleting the cells at 4°C. Cells were then resuspended in 50 μl of phosphate-buffered saline (PBS) containing 1% bovine serum albumin (BSA) and 1:200 diluted V450-conjugated rat anti-mouse CD107a (LAMP1). After 30 min on ice, the cells were washed with ice-cold PBS and analyzed by an LSRII flow cytometer (BD Biosciences). Median fluorescence intensities were determined and further processed using FlowJo software (Ashland).

### Cell activation

BMMCs were cultured for 48 hours in medium without SCF, followed by incubation in SCF- and IL-3-free medium supplemented with TNP-specific IgE (1 μg/ml). After 14 hours the cells were washed in buffered salt solution (BSS; 20 mM HEPES, pH 7.4, 135 mM NaCl, 5 mM KCl, 1.8 mM CaCl_2_, 5.6 mM glucose, 1 mM MgCl_2_) supplemented with 0.1% BSA and incubated with or without ethanol and/or other drugs. After 15 min, antigen was added and the cells were activated in the presence or absence of the drugs. The degree of degranulation was determined as the amount of β-glucuronidase released into the supernatant as described [32].

### Measurement of free intracellular calcium

Changes in concentrations of free intracellular calcium were determined using cell permeant Fura-2 acetoxymetyl ester (Fura-2 AM; Life Technologies) as a reporter. IgE-sensitized cells were harvested, washed in BSS-0.1% BSA and transferred to BSS-0.1% BSA supplemented with Fura-2 AM (1 ng/ml) and probenecid (2.5 mM) used to prevent dye leakage [33], and incubated in the shaker for 30 min at 37°C, 500 rpm. Fura-2 loaded cells were washed twice with 2.5 mM probenecid in BSS-0.1% BSA and then transferred to BSS-0.1% BSA supplemented with 2.5 mM probenecid and various drugs as specified in the Results. Cells were then incubated in Thermomixer (Eppendorf; 10 min, 37°C, 500 rpm). For measurement in the presence of extracellular calcium, cells were pelleted by centrifugation 500 x g for 3 min, resuspended in BSS-0.1% BSA and transferred to white polysorp 96 well plate (NUNC, Thermo Scientific). To determine free intracellular Ca^2+^ levels in the absence of extracellular Ca^2+^, cells were pelleted by centrifugation, washed in Ca^2+^-free BSS-0.1% BSA and transferred to the plate. Free intracellular Ca^2+^ was measured on INFINITE M200 (Tecan) as Fura-2 emission at 510 nm after excitation with 340 nm and 380 nm. Basal level of calcium was usually measured in the first 60 sec, followed by addition of antigen (TNP-BSA, final concentration 100 ng/ml) in BSS-0.1% BSA. Measurement continued up to 350 s. In the absence of extracellular calcium the cells were activated with antigen for 5 min followed by addition of CaCl_2_ to final concentration 1 mM and measurement continued up to 600 s.

### Immunoprecipitation and immunoblotting

Activated or nonactivated cells were solubilized in ice-cold lysis buffer (25 mM Tris-HCl, pH 8.0, 140 mM NaCl, 1 mM Na_3_VO_4_, 2 mM EDTA, 1 μg/ml aprotinin, 1 μg/ml leupeptin, 1 mM phenylmethylsulfonyl fluoride) supplemented with 0.2% Triton X-100 (for FcεRI immunoprecipitation). After incubation on ice for 30 min, the lysates were centrifuged (16,000 x g for 5 min at 4°C) and postnuclear supernatants were immunoprecipitated with rabbit anti-IgE antibody prebound to UltraLink-immobilized protein A (Pierce, Thermo Scientific). The immunoprecipitated proteins were size-fractionated by sodium dodecyl sulfate-polyacrylamide gel electrophoresis (SDS-PAGE), transferred to nitrocellulose membrane and immunoblotted with PY-20-HRP conjugate or with FcεRI-β chain-specific antibody followed by HRP-conjugated anti-mouse IgG. For immunoblotting analysis of other proteins, the cells were solubilized in lysis buffer supplemented with 1% n-dodecyl β-D-maltoside and 1% Nonidet P-40 and postnuclear supernatants were directly analyzed by SDS-PAGE followed by immunoblotting with phospho-protein-specific or the corresponding protein-specific antibodies and HRP-conjugated secondary antibodies. The HRP signals were detected by chemiluminescence, quantified by luminescent image analyzer LAS-3000 (Fuji Photo Film Co), and further analyzed by Aida image analyzer software (Raytest). The amount of phosphorylated proteins was normalized to the loading controls, run in parallel experiments [32]. We found that this approach gave more reliable data than normalization towards specific proteins after stripping of the membranes.

### Sucrose density gradient fractionation and expression of cytokine genes

Sucrose density gradient separations were performed as previously described [32] and individual fractions were analyzed by SDS-PAGE followed by immunoblotting with PY-20-HRP and protein-specific antibodies. Expression of cytokine genes was examined by quantitative polymerase chain reaction (qPCR) as described [32].

### Confocal microscopy

The wells of microscopy slides (CN Biomedicals) were coated with CellTak (BD Biosciences; 8 μl in 1 ml PBS). IgE-sensitized cells were left to attach to the coated wells for 15 min in BSS-0.1% BSA and then activated or not with Ag. After 15 min, the cells were fixed for 30 min with 3% paraformaldehyde in PBS and then permeabilized with 0.1% Triton X-100 in PBS for 30 min. After washing with PBS, the samples were blocked in PBS-1% BSA, and subsequently IgE was detected with Alexa Fluor 568-labeled donkey anti-mouse Ig. After labeling, the cells were washed and mounted in glycerol mounting solution supplemented with Hoechst 33258 stain to label nuclei. Samples were examined with a confocal laser scanning microscope Leica TCS SP5 equipped with an X63/1.4.N.A. oil-immersion objective. The image analysis was performed using a pipeline generated in CellProfiler software (Broad Institute, Boston) [34].

### ROS measurements

Reactive oxygen species (ROS) were examined with cell-permeant 2',7'-dichlorodihydrofluorescein diacetate (H_2_DCFDA; Life Technologies) as a reporter. Cells were sensitized for 16 hours with anti-TNP IgE (1 μg/ml) at 37°C in culture medium supplemented with 10% FCS, but devoid of SCF and IL-3. Then, the cells were washed and loaded into the wells of a 96-well plate (0.25 x 10^6^ cells/well) in 100 μl of the same medium supplemented with H_2_DCFDA (5 μg/ml) in the presence of probenecid (2.5 mM) and various concentrations of ethanol. After 15 min, the cells were centrifuged and resuspended in 50 μl BSS-BSA. Activation was triggered by adding 50 μl of 500 ng/ml TNP-BSA (final concentration 250 ng/ml) in the absence or presence of different concentrations of ethanol. Alternatively, in the experiment with methyl-β-cyclodextrin (Mβ) and cholesterol-saturated Mβ (sMβ), the cells were stained with H_2_DCFDA as above, washed and incubated 15 min in BSS-BSA supplemented or not with 0.5% (v/v) ethanol, 2 mM Mβ, and/or 2 mM sMβ. The activation was triggered by antigen (TNP-BSA; 250 ng/ml final concentrations) in the presence of the drugs; sMβ was prepared as previously described [35]. After 10 min, changes in fluorescence intensity were monitored in an LSRII flow cytometer (BD Biosciences; Ex/Em 488/505-535 nm).

### PCA

Mice were anesthetized by intraperitoneal injection of a cocktail containing ketamine (Narketan 10; 40 mg/kg, final concentration), xylazine (Xylapan; 10 mg/kg), and atropine (Atropin Biotika; 0.1 mg/kg) and then sensitized by intradermal injection of 20 μl of anti-TNP-specific IgE (50 μg/ml) in PBS into the left ear; the right ear was injected with 20 μl of PBS alone. Twenty-four hours later, 5% (v/v), 10% or 20% absolute ethanol in PBS was injected intraperitoneally (0.5 ml per mouse weighing 20 g). Mice injected with 0.5 ml of 10% or 20% ethanol exhibited reduced motoric activity, which lasted for 30 min or 90 min, respectively. Mice challenged with 0.5 ml of 5% ethanol were without any clinical signs. One hour after ethanol in PBS or PBS alone injection, each mouse was challenged with an intravenous injection of antigen (TNP-BSA, 100 μg) in 200 μl of PBS containing 1% Evans blue. The mice were killed 30 min later and their ears were removed for measurement of the amount of the Evans blue extravasated. The dye was extracted by adding 1 ml of formamide to each ear, followed by homogenization with the IKA T-25 ULTRA-TURRAX digital high-speed homogenizer systems (IKA) and incubation at 80°C. After 2 h, the formamide solution was centrifuged for 15 min at 13.000 x g and absorbance at 620 nm was determined in the supernatants.

### Statistical analysis

Statistical significance of intergroup differences was determined by one-way ANOVA with Tukey’s post-test using the Prism version 5.04 graphics and statistics software package (GraphPad); *, P<0.05; **, P<0.01, ***, P<0.001.

## Results

### Short-term exposure to ethanol inhibits FcεRI-induced degranulation and calcium response

**Fig 1:**
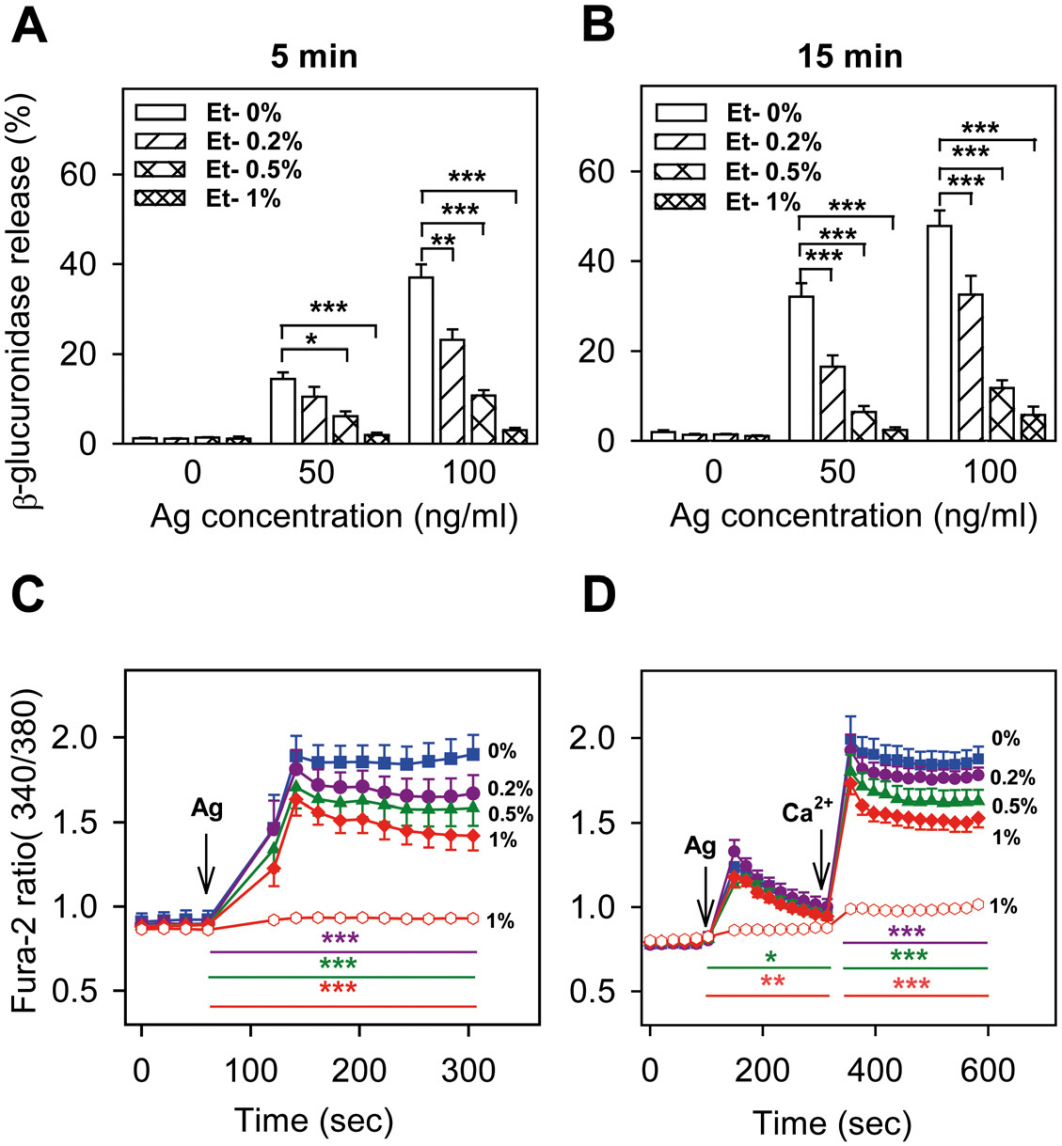
Short-term exposure to ethanol inhibits antigen-induced degranulation and calcium response in BMMCs. IgE-sensitized cells were preincubated for 15 min with various concentrations of ethanol (0–1%), which was also present during antigen-mediated activation. (A, B) Degranulation (release of β-glucuronidase) was measured 5 min (A) or 15 min (B) after exposure of the cells to the indicated concentrations of antigen. (C, D) Calcium response after addition of antigen (arrow, Ag, 100 ng/ml) was measured in the presence of 1 mM extracellular calcium (C), or in its absence (D), followed by addition of 1 mM calcium (arrow, Ca^2+^ in D). Calcium levels in the absence of antigen activation but in the presence of 1% ethanol is also shown in C and D (empty circles). Data are means ± SEs (n = 6–8). Statistical significance of intergroup differences is shown in A and B. In C and D, statistical significance of differences between control cells (0% ethanol) and cells exposed to 0.2% ethanol (violet line), 0.5% ethanol (green line) or 1% ethanol (red line) calculated for the corresponding time intervals (coloured lines) are also indicated.

To examine the inhibitory effect of ethanol on antigen-induced activation of mast cells, we isolated bone marrow cells from C57BL/6 mice and cultured them for 6–12 weeks in culture media supplemented with SCF and IL-3. More than 98% of the cells were mast cells as deduced from expression of both FcεRI and KIT receptors (see below). Exposure of BMMCs for 15 min– 2 hours with ethanol at concentrations up to 2% had no toxic effect, as determined by trypan blue dye exclusion test (not shown). Pretreatment of IgE-sensitized BMMCs for 15 min with ethanol at concentrations 0.2–1%, inhibited degranulation induced by multivalent antigen in a dose-dependent way. Significant inhibition was observed at all concentrations of ethanol tested when the cells were activated with antigen (50 ng/ml or 100 ng/ml) for 5 min or 15 min, except for cells treated with 0.2% ethanol and stimulated with antigen (50 ng/ml) for 5 min (Fig 1A and 1B).

**Fig 2:**
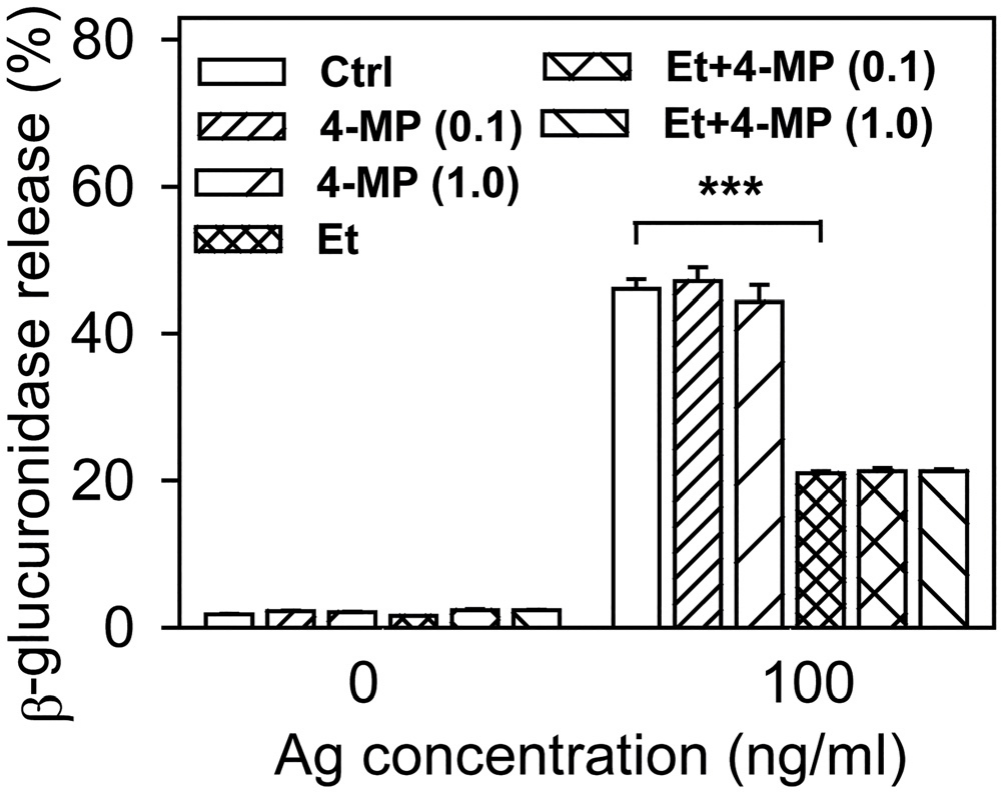
The inhibitory effect of ethanol on antigen-induced degranulation is not affected by blocking alcohol dehydrogenase. IgE-sensitized cells were pretreated or not (Ctrl) for 15 min with ethanol (0.5%) and/or 4-MP (0.1 or 1.0 mM). The cells were non-activated or activated with antigen (100 ng/ml) in the presence of ethanol and/or 4-MP for 15 min and degranulation was determined. Data are means ± SEs (n = 6). Statistical significance of differences between antigen-activated control cells and ethanol-treated cells is also shown. 4-

Ethanol at all concentrations used also inhibited, in a dose-dependent way, the calcium response after stimulation of IgE-sensitized cells with antigen in the presence of extracellular calcium (Fig 1C). When the cells were stimulated with antigen in the absence of extracellular calcium, the calcium response was significantly inhibited by 0.5% and 1% ethanol. Addition of extracellular calcium resulted in increased calcium response, which was significantly lower in cells exposed to 0.2%, 0.5% or 1% ethanol (Fig 1D). These data suggested that ethanol could act on early signaling events leading to the release of calcium from internal stores as well as on calcium channels directing influx of calcium from the extracellular space. When the cells were pretreated with 4-methylpyrazole (4-MP; 0.1 or 1 mM), a known inhibitor of alcohol dehydrogenase [36], the inhibitory effect of ethanol was not affected (Fig 2), suggesting that ethanol itself, rather than its metabolite product, is responsible for the inhibitory effect.

### Involvement of cholesterol in the inhibitory effect of ethanol on FcεRI-mediated activation

**Fig 3:**
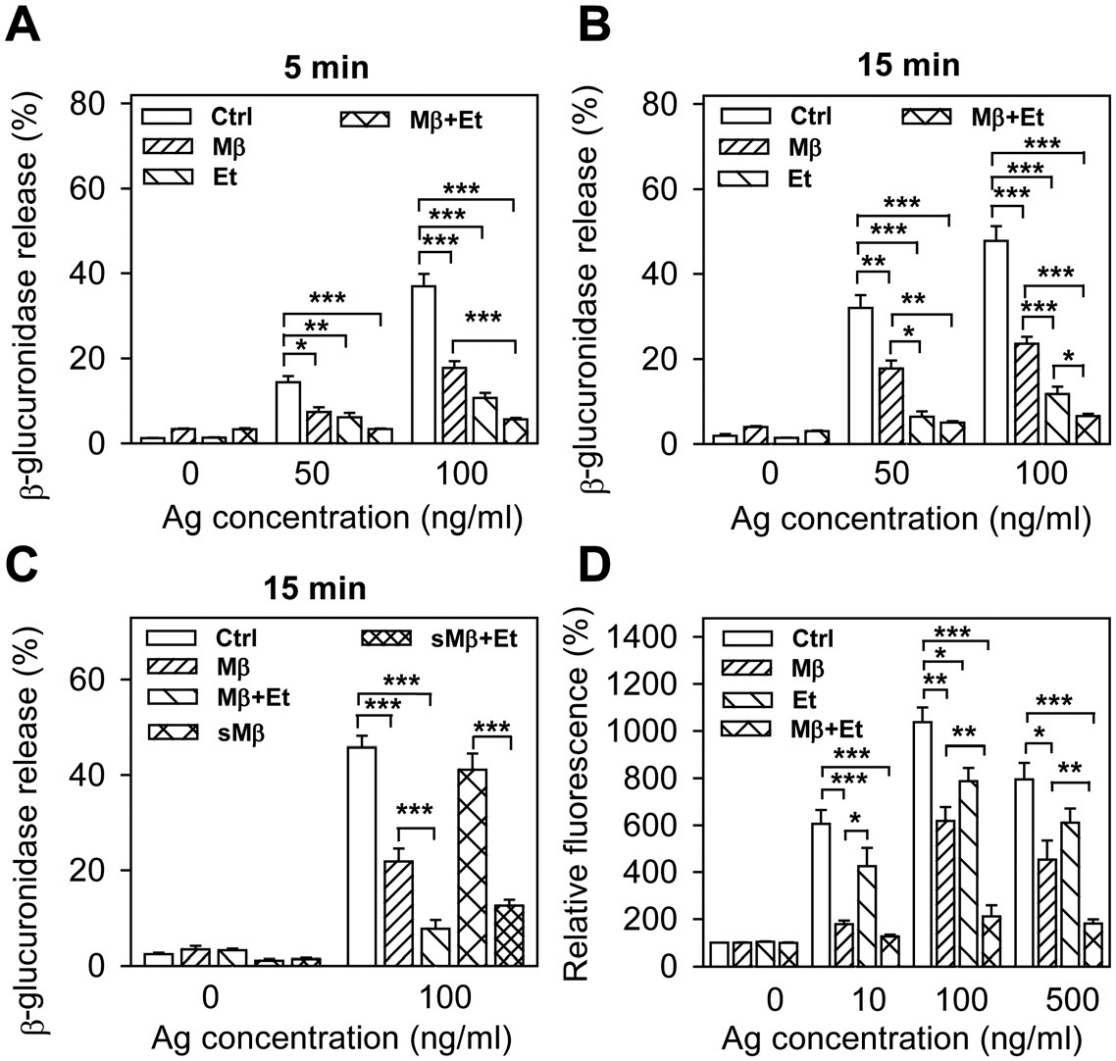
Involvement of cholesterol in the inhibitory effect of ethanol on FcεRI-mediated degranulation. (A, B) IgE-sensitized BMMCs were preincubated for 15 min without (Ctrl) or with Mβ (2 mM), and/or ethanol (0.5%), followed by exposure to the indicated concentrations of antigen in the presence of the drugs. Degranulation was measured 5 min (A) or 15 min (B) after triggering. (C) IgE-sensitized cells were preincubated for 15 min without (Ctrl) or with Mβ (2 mM), sMβ (2 mM), and/or ethanol (0.5%) and then activated (Ag; 100 ng/ml) or not in the presence of the drugs. Degranulation was determined 15 min after triggering. (D) IgE-sensitized cells were preincubated with various drugs as above, then exposed to various concentrations of antigen and cell activation, measured by CD107a (LAMP1) expression, was determined by flow cytometry. Data are means ± SEs (n = 4–12). Statistical significance of the intergroup differences is also shown.

Recently, Setiawan and Blanchard showed that ethanol interferes with the size and distribution of cholesterol domains in planar lipid bilayer structures [37]. To determine whether plasma membrane cholesterol could be involved in the inhibitory effect of ethanol, we evaluated the effect of Mβ alone or together with ethanol on FcεRI-mediated degranulation. Mβ is a water-soluble cyclic heptasaccharide that binds cholesterol and can extract cholesterol from the exoplasmic leaflet of the plasma membrane by harboring cholesterol in a hydrophobic cavity [35,38,39]. Exposure of cells for 15 min to 2 mM Mβ alone or in combination with 0.5% ethanol had no significant effect on spontaneous release of β-glucuronidase (Fig 3A–3C). In cells activated with antigen at a concentration 50 ng/ml or 100 ng/ml for 5 (Fig 3A) or 15 min (Fig 3B), degranulation was significantly reduced by both Mβ and ethanol. When ethanol and Mβ were used together, the inhibition of degranulation was higher than in cells treated with ethanol or Mβ alone. Mβ is not specific for cholesterol, but has pleiotropic effects on the level and distribution of various membrane components [40]. Next, we therefore evaluated antigen-induced degranulation in cells pretreated with cholesterol-saturated Mβ (sMβ), which increases plasma membrane cholesterol [35]. We found that sMβ, in contrast to Mβ, had no significant inhibitory effect on degranulation (Fig 3C). Interestingly, sMβ-treatment did not protect the cells from the inhibitory effect of ethanol (Fig 3B and 3C). We also found that exposure of cells to 2 mM Mβ or sMβ with or without ethanol for 15 min had no effect on the FcεRI expression as determined by flow cytometry (not shown).

Degranulation is accompanied by enhanced surface expression of CD107a (LAMP1), a secretory granule/lysosomal marker detectable by flow cytometry [41]. In agreement with antigen-induced β-glucuronidase release, 2 mM Mβ and 0.5% ethanol alone inhibited CD107a (LAMP1) surface expression, even though ethanol was less potent. An additive effect of the drugs was observed when the cells were activated with antigen at optimal (100 ng/ml) and supra-optimal (500 ng/ml) concentrations (Fig 3D).

**Fig 4:**
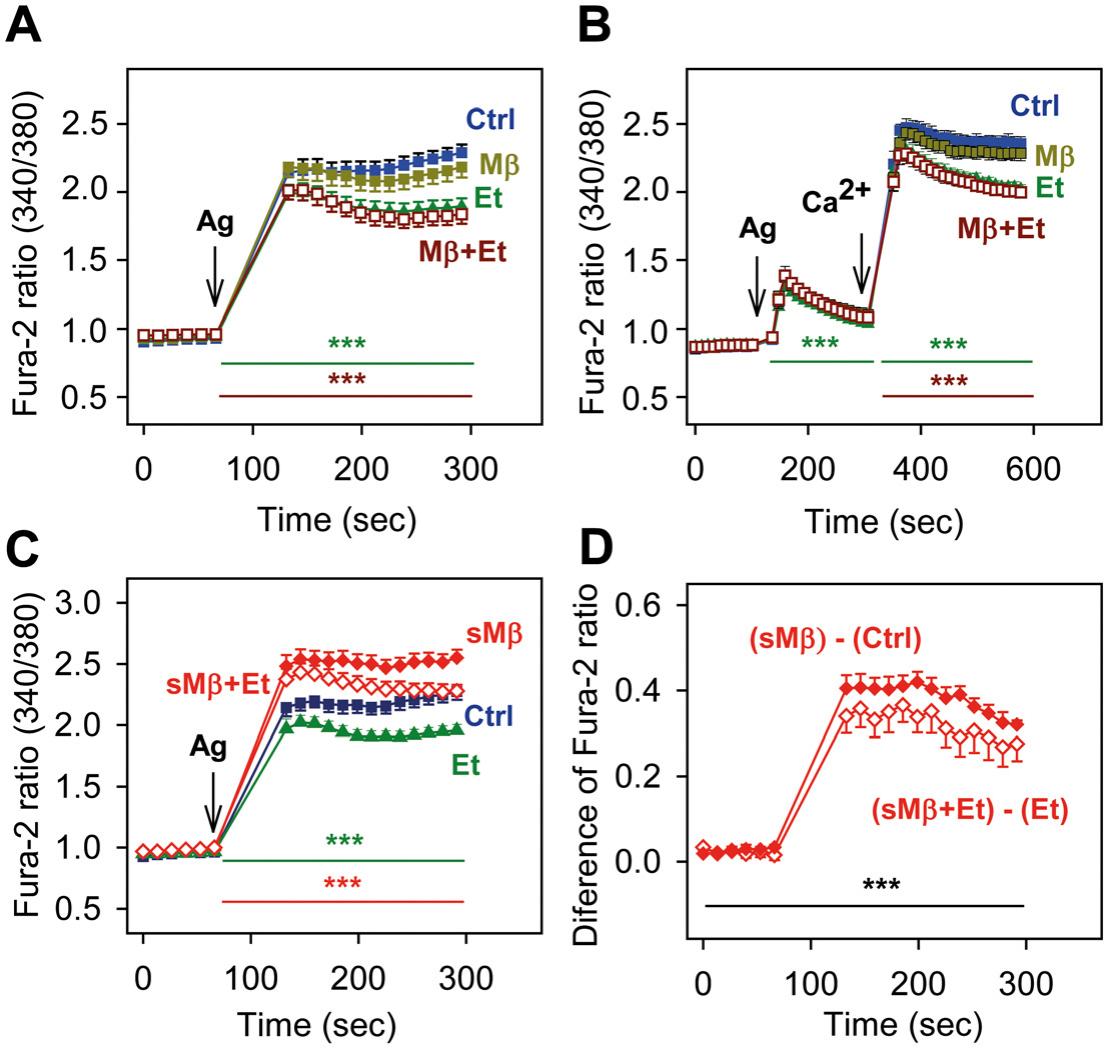
Involvement of cholesterol in the inhibitory effect of ethanol on FcεRI-mediated calcium response. (A, B) IgE-sensitized BMMCs were incubated for 15 min without (Ctrl) or with ethanol (Et, 0.5%) and/or Mβ (2 mM). Calcium response after adding antigen (arrow, Ag; 100 ng/ml) was measured in the presence of the drugs as indicated. The cells were activated in the presence of extracellular calcium (A) or in its absence, followed by addition of 1 mM calcium (B, Ca^2+^, arrow). (C) IgE-sensitized cells were incubated for 15 min in medium alone (Ctrl) or medium supplemented with ethanol (Et; 0.5%), Mβ saturated with cholesterol (sMβ; 2 mM), or sMβ (2mM) and ethanol (0.5%). The cells were activated in the presence of the drugs with antigen (arrow, Ag; 100 ng/ml) and calcium response was determined. (D) Data from Fig 4C were used to calculate the difference between calcium response in cells exposed to sMβ or Ctrl and sMβ+Et or Et. Data are means ± SEs (n = 6–12). Statistical significance of differences in A and B [Ctrl versus Et-treated cells (green line) and Ctrl versus Mβ+Et-treated cells (brown line)], C [sMβ versus Ctrl (green line) and sMβ+Et versus Et (red line)], and D [sMβ—Ctrl versus sMβ+Et—Et (black line)] calculated for the corresponding time intervals (coloured lines) are also indicated.

We also analyzed the combined effect of Mβ and/or ethanol pretreatment on antigen-induced calcium response. The data indicate that pretreatment with Mβ did not intensify the inhibitory effect of ethanol (Fig 4A). When the cells were activated in the absence of extracellular calcium, the inhibitory effect of ethanol was abrogated by Mβ. After addition of calcium, both ethanol and ethanol+Mβ showed comparable responses (Fig 4B). Cells pretreated with sMβ showed increased calcium response and were more resistant to the inhibitory effect of ethanol (Fig 4C). When the difference between sMβ + ethanol and ethanol was calculated, it was lower than the difference between sMβ and controls (Fig 4D). The data suggest that manipulation with the cellular cholesterol modulates sensitivity of antigen-induced calcium response to ethanol, and thus support the lipid-centric hypothesis of ethanol action in this system.

### Ethanol exposure or cholesterol removal inhibit expression of cytokine genes in antigen-activated BMMCs

**Fig 5:**
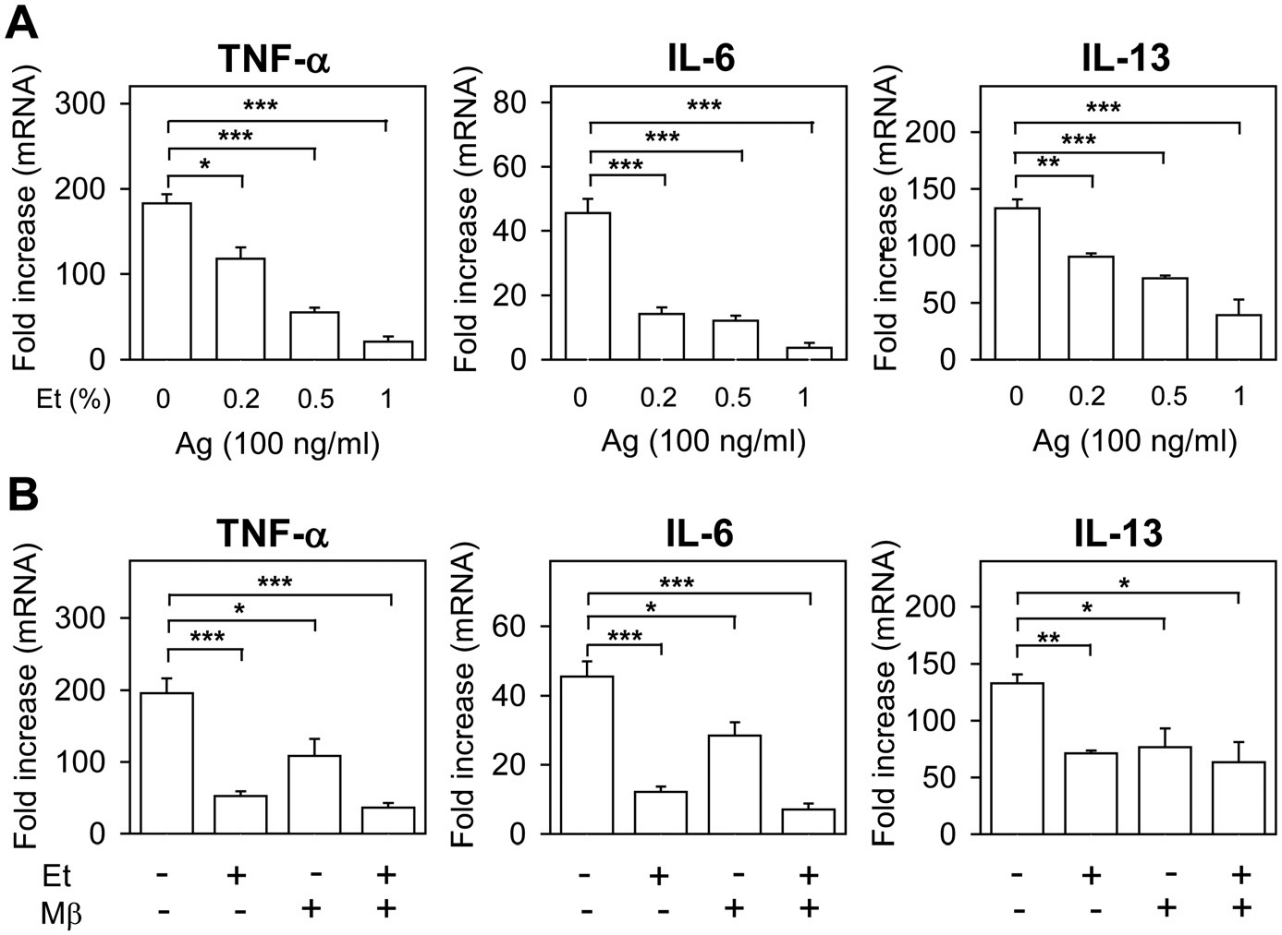
Ethanol or cholesterol removal inhibit expression of cytokine genes in antigen-activated BMMCs. (A) IgE-sensitized cells were preincubated for 15 min with the indicated concentrations of ethanol, which were also present during activation with antigen (100 ng/ml). mRNAs for TNF-α, IL-6, and IL-13 were isolated one hour after triggering and quantified by qPCR. (B) The cells were exposed to medium alone (-), ethanol (0.5%) and/or Mβ (2 mM) and mRNAs for TNF-α, IL-6, and IL-13 were quantified as above. Data are means ± SEs (n = 6–8). The statistical significance of the intergroup differences is also shown.

Next we examined the effect of short-term pretreatment of BMMCs with various concentrations of ethanol (0–1%) on antigen-induced expression of cytokines. Data presented in Fig 5A indicate dose-dependent inhibition of antigen-induced mRNA production of TNF-α, IL-6 and IL-13, as determined by qPCR. Transcriptional inhibition of cytokines was also observed in cells with reduced levels of cholesterol after pretreatment with Mβ. When the cells were pretreated simultaneously with both Mβ and ethanol, less-than-additive effects were observed (Fig 5B). These data suggest that both drugs could inhibit a similar signaling pathway.

### Protective effect of cholesterol against ethanol-mediated inhibition of ROS in antigen-activated BMMCs

**Fig 6:**
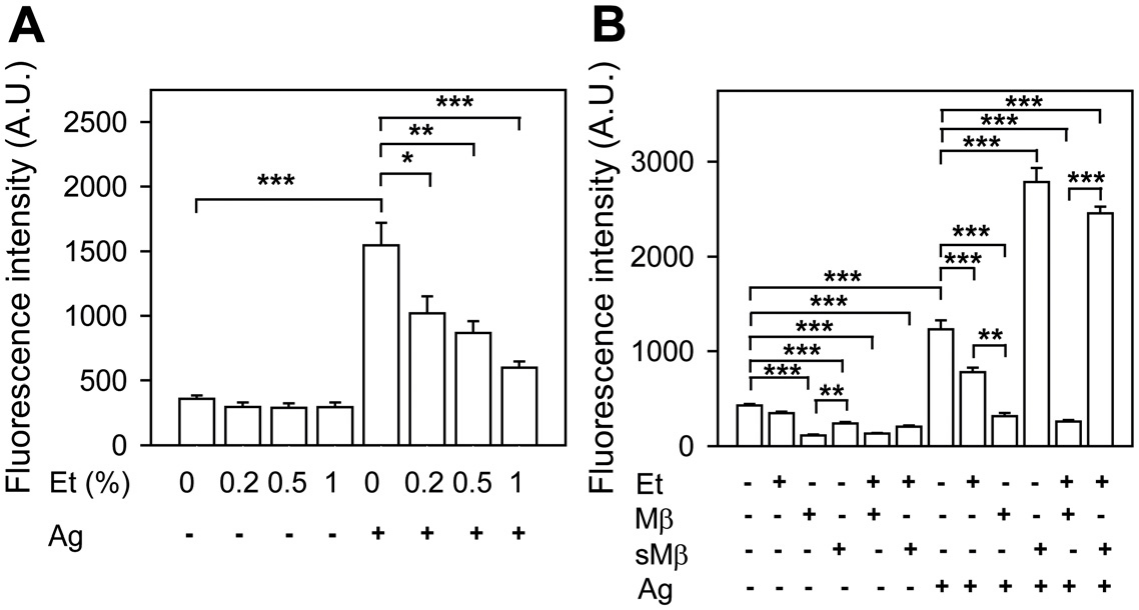
Protective effect of cholesterol against ethanol-mediated inhibition of ROS production in antigen-activated BMMCs. (A) IgE-sensitized cells were incubated for 15 min with the indicated concentrations of ethanol, which was also present during the activation. Then the cells were activated or not with antigen (250 ng/ml) and ROSs were determined using H_2_DCFDA as a substrate. The values on y-axes indicate fluorescence intensities observed 10 min after triggering. (B) The cells were exposed to BSS-BSA supplemented or not with ethanol (0.5%), Mβ (2 mM) and/or sMβ (2 mM), and after 20 min activated or not with antigen (250 ng/ml). ROSs were determined as above. Data are means ± SEs (n = 6–8). The statistical significance of the intergroup differences is also shown.

Recent studies indicated that exposure of macrophages to ethanol promoted generation of ROS [42–44]. To determine whether ROS could also be generated by ethanol in BMMCs, we first measured ROS production in cells exposed to various concentrations of ethanol. Our data show that short-term exposure to ethanol had no effect on production of ROS in non-activated cells (Fig 6A). In cells activated with antigen, in accord with previous studies [45,46], ROSs were increased. Pretreatment with ethanol resulted in a dose-dependent decrease of ROS after activation with antigen (Fig 6A). Next we examined whether cholesterol could be involved in ROS production (Fig 6B). IgE-sensitized cells were pretreated or not with ethanol, Mβ, and/or sMβ. In non-activated cells, ethanol had no significant effect on ROS, but pretreatment with Mβ reduced baseline ROS production. sMβ had a lower inhibitory effect on ROS levels than Mβ. In antigen-activated cells, ROS production was more inhibited by Mβ than by ethanol. In contrast, pretreatment with sMβ followed by antigen activation resulted in higher ROS production than in control cells. When ethanol was combined with Mβ, strong inhibition of antigen-induced ROS production was observed, whereas ethanol had no inhibitory effect in cells exposed to sMβ. These data suggest that cholesterol has a dramatic effect on ROS production and its sensitivity to ethanol.

### Inhibitory effect of ethanol on tyrosine phosphorylation of FcεRI-related signal transduction proteins

**Fig 7:**
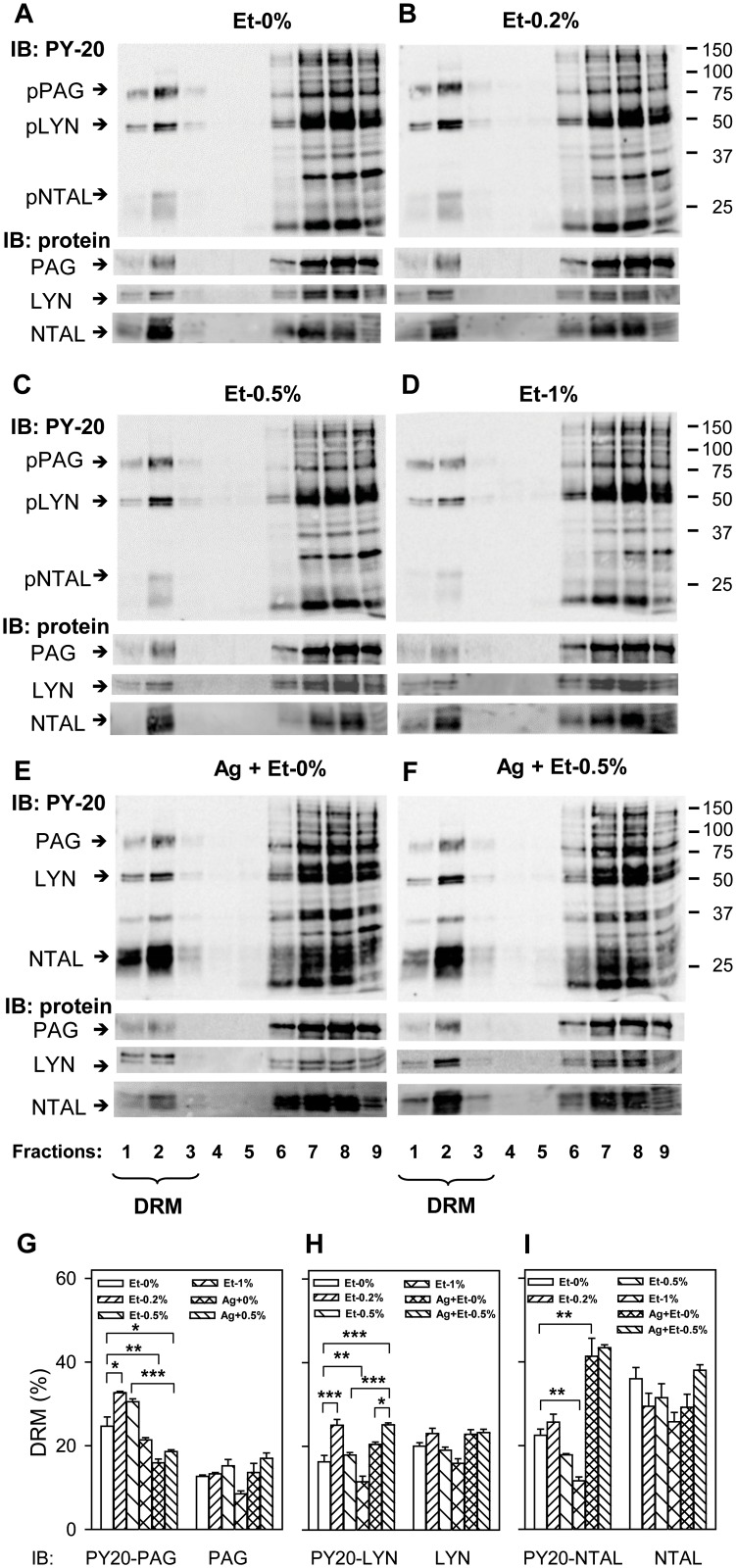
Ethanol-induced changes in protein tyrosine phosphorylation in the plasma membrane domains. (A–F) IgE-sensitized BMMCs were pretreated or not with the indicated concentrations of ethanol for 15 min and then non-activated (A–D) or activated with antigen (E and F; 100 ng/ml) for 5 min. Then the cells were solubilized in 1% Brij-96-containing lysis buffer and fractionated on sucrose density gradient. Individual fractions were collected from the top of the gradient (fraction 1), size fractionated by SDS-PAGE and examined for tyrosine phosphoproteins by immunoblotting (IB) with PY-20-HRP conjugate (PY-20) or with antibodies specific for PAG, LYN, and NTAL). Positions of PAG, LYN, and NTAL are indicated by arrows on the left. Fractions (1–3) containing detergent-resistant membranes are marked (DRM). Numbers on the right indicate positions of molecular weight markers in kDa. Representative immunoblots from three to four independent experiments are shown. (G–I) All immunoblots were analyzed by densitometry, and the relative amounts of PAG (G), LYN (H), and NTAL (I) and their tyrosine phosphorylated forms (PY-20) in DRMs were determined. Means ± S.E. were calculated and the statistical significance of intergroup differences was determined.

The first biochemically well-defined step in FcεRI signaling is tyrosine phosphorylation of the receptor subunits, followed by formation of the FcεRI signalosome and phosphorylation of other substrates [47]. Next, we therefore investigated global tyrosine phosphorylation of proteins in non-activated or antigen-activated cells pretreated or not with various concentrations of ethanol. To determine whether there are any changes in association of the tyrosine phosphorylated proteins with membrane domains, the cells were solubilized in solubilization buffer containing 1% Brij-96 and fractionated on sucrose density gradient to separate cellular components according to their solubility in detergents and density. Data presented in Fig 7A indicate distribution of tyrosine phosphorylated proteins in non-activated control cells, not exposed to ethanol. In low-density detergent-resistant membranes (DRM, fractions 1–3), the key tyrosine phosphorylated proteins were transmembrane adaptor proteins PAG and NTAL and SRC family kinase LYN, as determined by immunoblotting with protein-specific antibodies. A number of other tyrosine phosphorylated proteins were observed in detergent-soluble fractions (fractions 6–9). In cells pretreated with various concentrations of ethanol (Fig 7B–7D), there were dose-dependent changes in the distribution of some tyrosine phosphorylated proteins in the low-density and high-density fractions. Densitometry analysis of the immunoblots showed a significant increase in the percentage of tyrosine phosphorylated PAG (Fig 7G) and LYN (Fig 7H) in DRMs from cells exposed to low concentrations of ethanol (0.2%). However, when ethanol was used at a higher concentration (1%), percentage of tyrosine phosphorylated LYN (Fig 7H) and NTAL (Fig 7I) in DRMs was significantly reduced (Fig 7D). In antigen-activated cells, some proteins were more tyrosine phosphorylated in both low- and high-density fractions (compare Fig 7A and 7E). After activation, the percentage of tyrosine phosphorylated LYN in DRMs was increased by ethanol (Fig 7H). When PAG-, LYN-, or NTAL-specific antibodies were used, no significant differences in the distribution of proteins in DRMs from control and ethanol-pretreated cells were observed (Fig 7G–7H). These data suggest that ethanol at the concentrations used caused fine changes in the cross-talk between signaling molecules rather than global changes in the detergent solubility of plasma membrane proteins.

**Fig 8:**
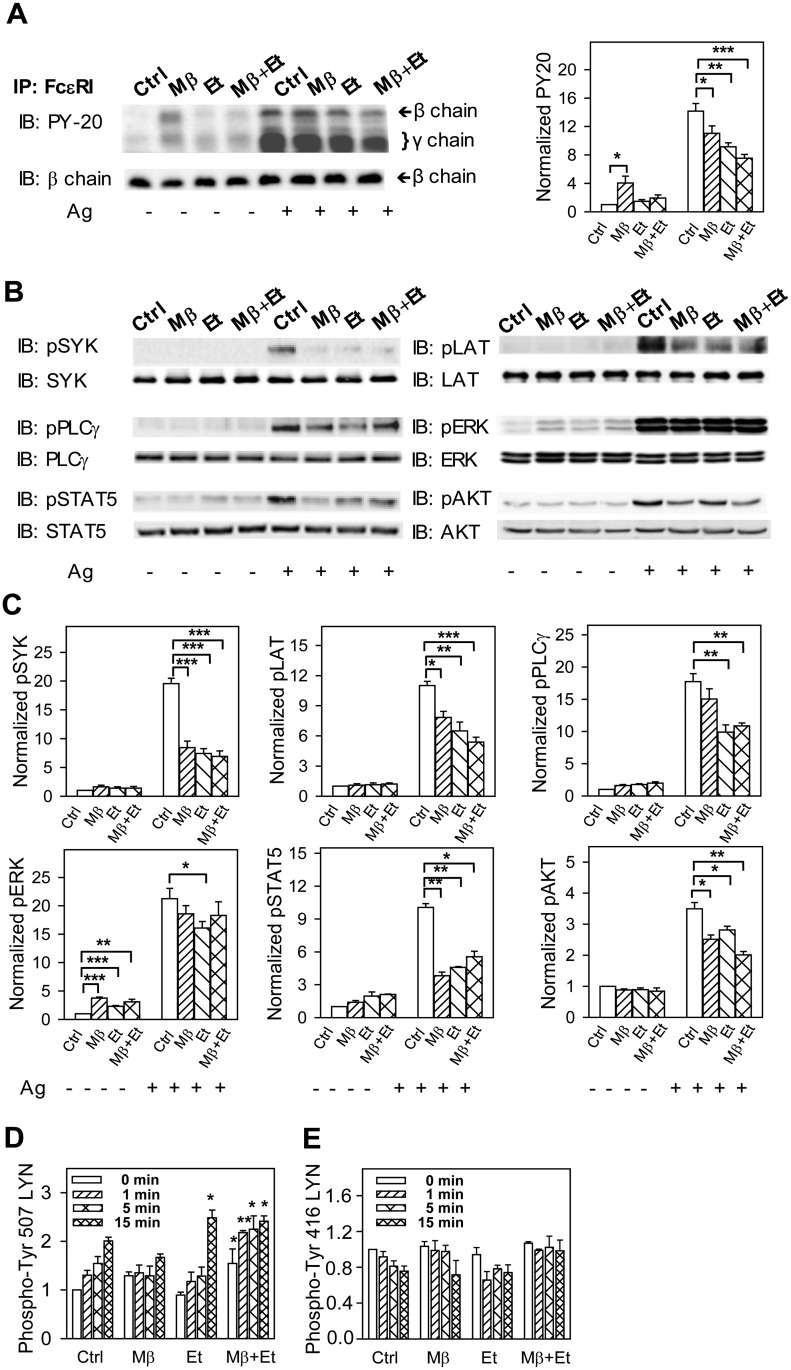
Pretreatment with ethanol inhibits tyrosine phosphorylation of FcεRI β and γ subunits and some other proteins involved in FcεRI signaling. (A) IgE-sensitized cells were preincubated for 15 min with BSS-BSA alone (Ctrl) or supplemented with ethanol (0.5%) and/or Mβ and then activated or not with antigen (100 ng/ml) in the presence or absence of the compounds. After 5 min the cells were solubilized in 0.2% Triton X-100 and FcεRI was immunoprecipitated (IP) from postnuclear supernatants. The immunoprecipitates were resolved by SDS-PAGE and analyzed by immunoblotting with PY-20-HRP conjugate. For loading controls, the same membrane was stripped and re-blotted with FcεRI-β-chain-specific antibody. Representative immunoblots from three to five independent experiments are shown on the left. The immunoblots were analyzed by densitometry and the fold increase in tyrosine FcεRI-β and -γ chain phosphorylation, normalized to non-activated cells and the amount of FcεRI-β chain, is also shown on the right. (B) IgE-sensitized cells were incubated and activated as above. Five min after triggering the cells were solubilized, size fractionated, and tyrosine phosphorylated proteins were detected by immunoblotting with the phosphoprotein-specific antibodies. Antibodies for the corresponding proteins were used for detection of loading controls. Representative immunoblots from three to four independent experiments are shown. (C) The immunoblots were analyzed by densitometry. Fold increases of protein tyrosine phosphorylation, normalized to control (Ctrl) non-activated cells and the corresponding protein loads are shown. (D and E) IgE-sensitized cells were incubated with the drugs as in A and then activated with antigen (100 ng/ml) in the presence of the drugs for the indicated time intervals. The cells were solubilized, size fractionated, and LYN phosphorylated on Tyr 507 (D) or Tyr 416 (E) was detected by immunoblotting with the corresponding antibodies. After stripping, the membranes were developed for LYN used as a loading control. Fold increase in protein tyrosine phosphorylation, normalized to non-activated cells (Ctrl) and protein load, is also shown. Means ± SEs and the statistical significance of differences in A, C, E, and D were calculated from three to five independent experiments.

To determine changes in tyrosine phosphorylation of FcεRI, the receptor was immunoprecipitated and analyzed by immunoblotting with phosphotyrosine-specific antibody, PY-20-HRP conjugate. In non-activated cells, the receptor β and γ subunits exhibited weak tyrosine phosphorylation, and ethanol (0.5%) with or without Mβ had no significant effect on their phosphorylation (Fig 8A). In cells exposed to Mβ alone, a significant increase in tyrosine phosphorylation of FcεRI β and γ subunits was observed, which is in line with our previous studies indicating that cholesterol removal enhances phosphorylation of various substrates in mast cells [48]. Tyrosine phosphorylation of FcεRI β and γ subunits was increased after FcεRI triggering, as expected [47], and pretreatment with Mβ, ethanol, and especially Mβ+ethanol, significantly reduced this phosphorylation (Fig 7A).

Tyrosine phosphorylation of several other proteins involved in FcεRI signaling was examined by direct immunoblotting of size-fractionated cell lysates with phosphotyrosine protein-specific antibodies. For these experiments we used IgE-sensitized cells pretreated or not with Mβ and/or ethanol (Fig 8B and 8C). The cells were either non-activated or activated with antigen for 5 min. For these studies we selected proteins involved in tyrosine phosphorylation of the FcεRI subunits (LYN and SYK), regulation of calcium response (LAT and PLCγ), and transcriptional regulation of cytokines (STAT5, ERK, AKT). We found that in non-activated cells, Mβ alone and/or ethanol either had no effect (SYK, LAT, pLCγ1, STAT5, and AKT) or slightly but significantly increased phosphorylation of the target (ERK). In antigen-activated cells, pretreatment with Mβ alone and/or ethanol reduced tyrosine phosphorylation of several target proteins (SYK, LAT, STAT5, and AKT). When pPLCγ1 was analyzed, only ethanol and Mβ+ethanol showed inhibitory effects, whereas in the case of pERK, only ethanol alone was inhibitory.

Activity of mouse LYN kinase is regulated by tyrosine phosphorylation of Tyr 507 (a negative regulator; Fig 8D) and Tyr 416 (a positive regulator; Fig 8E). To determine whether Mβ and/or ethanol interfere with phosphorylation of these tyrosines, we performed immunoblotting with LYN-tyrosine-specific antibodies. We found that pretreatment of cells with 0.5% ethanol enhanced phosphorylation at LYN-Tyr 507 15 min after FcεRI triggering. Mβ together with ethanol increased phosphorylation of LYN-Tyr 507 at all intervals. In contrast, LYN-Tyr 416 did not exhibit any significant changes in phosphorylation after Mβ and/or ethanol exposure and antigen activation. These data suggest that ethanol could inhibit FcεRI activation by enhancing tyrosine phosphorylation of LYN-Tyr 507.

### Pretreatment with ethanol and/or cholesterol removal does not interfere with FcεRI expression but affects its internalization

**Fig 9:**
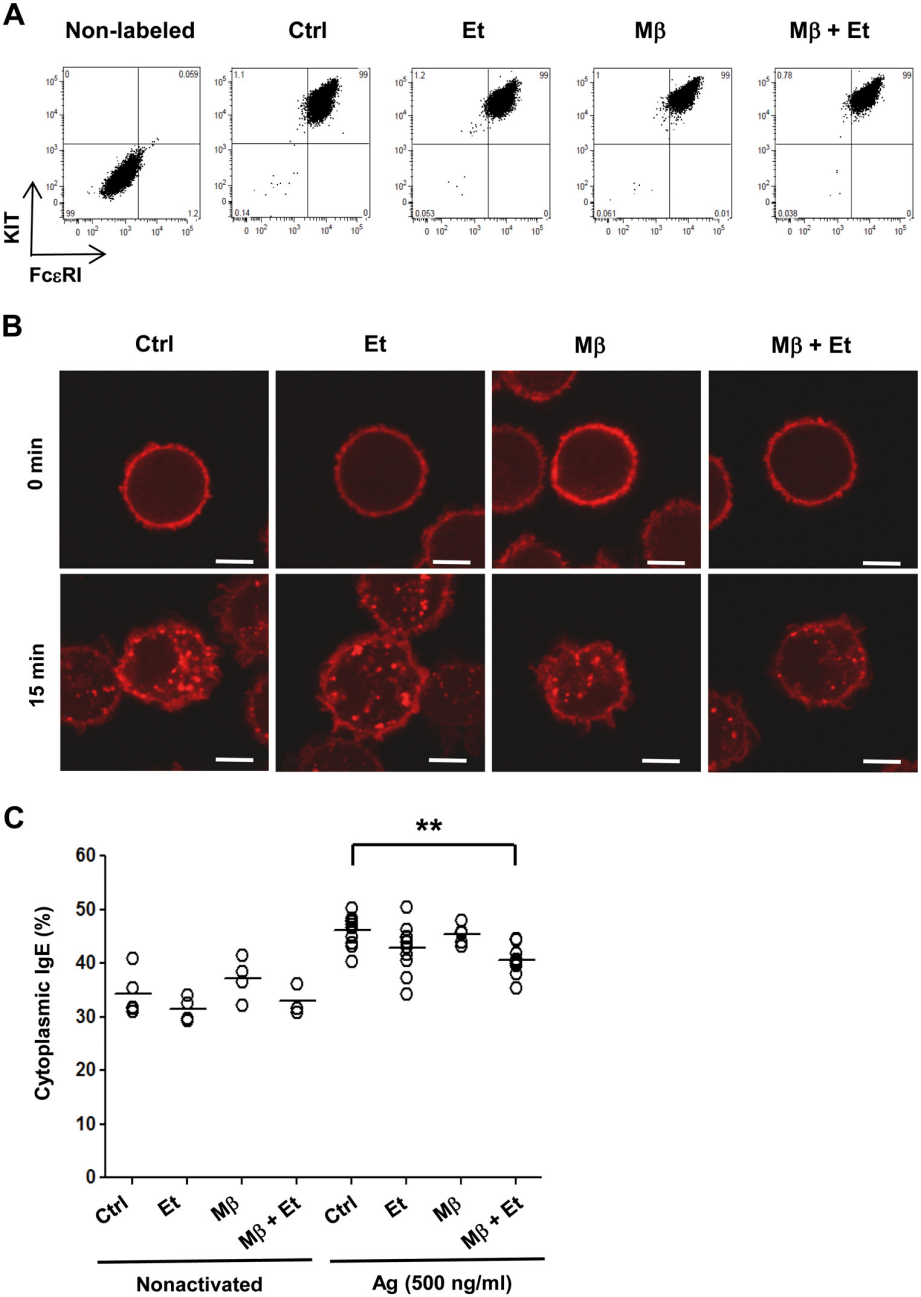
Pretreatment with ethanol and cholesterol removal do not interfere with the FcεRI expression but affect its internalization. (A) The cells were incubated or not for 15 min with BSS-BSA alone (Ctrl) or supplemented with ethanol (0.5%) and/or Mβ (2 mM) and then stained for surface KIT and FcεRI by direct immunofluorescence followed by flow cytometry analysis. (B) IgE-sensitized cells were incubated with the drugs as above and activated or not with antigen (500 ng/ml). After 15 min the cells were fixed, permeabilized, labeled for IgE and analyzed by confocal microscopy. Bars = 5 μm. (C) Distribution of IgE in individual cells was evaluated and the fraction of IgE detected in the cytoplasm was determined. Each spot represents one cell, bars indicate means. Statistical significance of intergroup differences is also indicated.

Previous studies with other cell types showed that ethanol interferes with internalization of plasma membrane receptors, their trafficking, endocytosis, and recycling [49–51]. FcεRI is rapidly internalized upon antigen triggering [52,53]. Next, we therefore compared internalization of antigen-aggregated FcεRI in BMMCs pretreated or not with ethanol and/or Mβ. Data presented in Fig 9A indicate that 15 min pretreatment with 0.5% ethanol and/or 2 mM Mβ had no effect on the surface expression of the FcεRI and KIT. When the cells were pretreated with ethanol and/or Mβ and then permeabilized and stained for total IgE, no significant differences in the amount of internalized IgE were determined in non-activated cells. In cells activated with antigen for 15 min, IgE was internalized, and pretreatment with ethanol together with Mβ significantly reduced the internalization. Pretreatment with ethanol or Mβ alone had no significant effect on internalization of the antigen-IgE-receptor complexes (Fig 9B and 9C).

### Ethanol suppresses IgE-mediated PCA in mice

**Fig 10:**
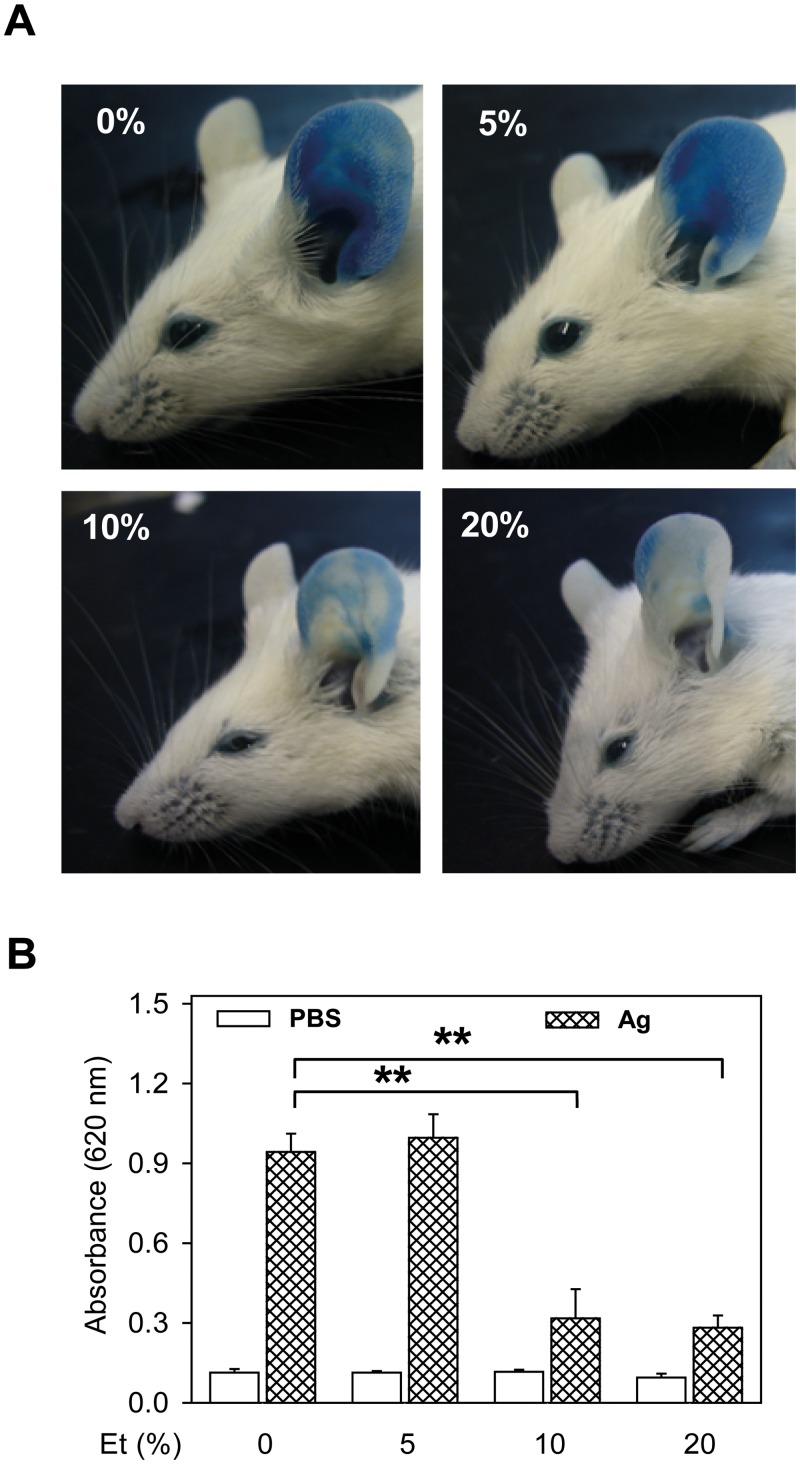
Inhibitory effect of ethanol on mast cell-mediated PCA. PCA was performed as described in Materials and methods. Sensitizing IgE in PBS and PBS alone were injected into left and right ears, respectively. (A) Representative photographs of ears of the mice injected intraperitoneally with 0.5 ml (per mouse weighing 20 g) PBS alone (0%) or with 0.5 ml of PBS containing 5%, 10%, or 20% ethanol, followed by intravenous administration of Evans blue and antigen in PBS. (B) Quantitative data for ear-tissue extracted Evans blue from left (IgE) and right (PBS) ears in mice treated as above. Means ± SEs were calculated from 3–4 animals in each group. Statistically significant differences between control mice injected with PBS alone and mice injected with 10% or 20% ethanol in PBS are shown.

Finally, we examined whether ethanol could have an inhibitory effect on mast cells under *in vivo* conditions. We used PCA in which local activation of mast cells results in increased vascular permeability, as visibly manifested by leakage of the Evans blue dye into the reaction site of the ear. This leakage was not affected by intraperitoneal administration of 0.5 ml PBS containing 5% ethanol (Fig 10A and 10B; compare 0% and 5%) 30 min before the antigen. In contrast, in mice that were injected with 10% or 20% ethanol in PBS, the vascular permeability of the ears was attenuated, as evaluated by the extent of Eva blue staining (Fig 10A, 10% and 20%) and amounts of Evans blue extracted from the ears (Fig 10B). The difference in PCA between mice injected with 0.5 ml PBS alone or 0.5 ml PBS containing 10% or 20% ethanol was significant (Fig 10B). No significant intergroup differences were noticed when control ears (not sensitized with IgE) were evaluated. The data suggest that ethanol inhibits FcεRI-induced mast cell activation *in vivo*.

## Discussion

Data presented in this study show that short-term exposure of BMMCs to nontoxic concentrations of ethanol inhibits FcεRI-mediated degranulation, calcium response, and production of several cytokines (TNF-α, IL-6, and IL-13) in a dose-dependent manner. To understand the molecular mechanism of the inhibitory effect of ethanol on these activation events we examined various candidate targets. Several lines of evidence suggest that ethanol interferes with the function of FcεRI-cholesterol signalosomes and support the lipid-centric theory of ethanol action in this system, at least at the early stages of cell activation

First, pretreatment of the cells with 4-MP, an inhibitor of alcohol dehydrogenase, together with ethanol had no effect on the inhibitory action of ethanol on degranulation. Thus, inhibition of mast cell activation seems to be caused by ethanol itself and not its metabolites. This conclusion is supported by the finding that up to 2 hours exposure to 2% ethanol in BSS-BSA at 37°C was not toxic to BMMCs. It should also be noted that the cells in this study were pretreated with ethanol only for 15 min before activation, which makes less likely that metabolites are formed and affect signaling pathways.

Second, antigen-mediated degranulation, calcium response, and production of cytokines were also inhibited by Mβ. In cells treated with Mβ, the inhibitory effect of ethanol on degranulation, and IgE receptor internalization was potentiated. Interestingly, when the cells were pretreated with Mβ saturated with cholesterol, the inhibitory effect of ethanol on calcium response was reduced. These data suggest that cholesterol is involved in the inhibitory effect of ethanol.

Third, production of ROS after FcεRI triggering was also inhibited by ethanol in a dose-dependent manner and treatment together with Mβ potentiated the inhibitory effect of ethanol. These findings are in accord with previous data showing that lowering cellular cholesterol by Mβ in hepatocytes inhibits ROS production [54]. Interestingly, exposure of the cells to sMβ enhanced ROS production, and the inhibitory effect of ethanol was abolished, confirming that cholesterol interferes with the inhibitory activity of ethanol.

Fourth, immunoprecipitation and immunoblotting analyses showed that ethanol inhibits tyrosine phosphorylation of FcεRI β and γ subunits in antigen-activated cells. Phosphorylation of these targets is the first biochemically well-defined step after FcεRI triggering and depends on the topography and activity of protein tyrosine kinase LYN and protein tyrosine phosphatases in the vicinity of the FcεRI [55,56]. The impaired tyrosine phosphorylation of the FcεRI β and γ subunits could explain reduced tyrosine phosphorylation of the downstream substrates, including SYK, LAT, PLCγ, AKT, and STAT5 in ethanol-treated cells. Tyrosine phosphorylation of these substrates was also inhibited by Mβ, but no clear additive effect of ethanol and Mβ was observed. This could be related to a similar mechanism of inhibition of both drugs. The reduced phosphorylation of FcεRI by ethanol was neither caused by reduced phosphorylation of the positive regulatory Tyr 416 of LYN nor by enhanced phosphorylation of the negative regulatory Tyr 507 of LYN, which showed enhanced phosphorylation only 15 min after activation. Surprisingly, when Mβ and ethanol were used together, enhanced Tyr 507 phosphorylation was observed at all time intervals after activation and even in non-activated cells. Tyr 507 is phosphorylated by CSK, which is presumably anchored to the plasma membrane via phosphorylated PAG [32]. However, our study did not show enhanced tyrosine phosphorylation of PAG in the cells pretreated with ethanol. The enhanced phosphorylation of Tyr 507 of LYN could be related to our recent findings that PAG is both a positive and negative regulator of FcεRI signaling and that in mast cells there are some other not yet discovered anchors of CSK [32]. Enhanced phosphorylation of LYN Tyr 507 in cells pretreated with ethanol alone, but not in cells treated with Mβ alone, was not accompanied by the corresponding inhibition of SYK phosphorylation, which was observed in cells pretreated with both ethanol alone and Mβ alone. Thus, rather than changes in activity of the LYN kinase, ethanol and Mβ could interfere with formation of the FcεRI signalosome in which cholesterol could play a key role and in which kinases and phosphatases are at equilibrium in nonactivated cells [55]. Nanoscale changes in lateral organization of proteins and lipids in the plasma membrane and enhanced actin polymerization have recently been described in ethanol-pretreated cells [57]. In fact, there could be a direct cross-talk between ethanol and cholesterol, as was noticed in other systems. For example, Furlow and Diamond showed that the interplay between membrane cholesterol and ethanol contributes to alterations of the membrane fluidity, viscosity, and redistribution of surface molecules, which affects neutrophil adhesion, rolling, and tethering behavior [58].

Fifth, internalization of antigen-aggregated FcεRI was significantly inhibited by 15 min pretreatment with Mβ and ethanol. In our previous study we found that cholesterol removal by Mβ enhanced antigen-mediated clustering of the FcεRI [48]. These findings, together with a previous report that aggregated FcεRI can be endocytosed by a clathrin-independent mechanism that appears to be mediated by membrane structures enriched in cholesterol [52], suggest that Mβ and ethanol prevent internalization of the receptor aggregates, which remain for prolonged time intervals on the cell surface.

The capacity of externally added cholesterol to reduce the inhibitory effect of ethanol on calcium and ROS responses are more compatible with the lipid-centric theory than the protein-centric theory of the inhibitory effect of ethanol. How exactly ethanol interacts with lipids and/or how it affects lipid-protein interactions is not known but could involve direct interaction of ethanol with cholesterol [59] and the ability of ethanol to mediate and modify organization of the lipid bilayer structures [7,37]. However, our data do not rule out the possibility that ethanol-protein interactions are also involved in the inhibitory effect of ethanol as was observed in other systems [12,60–62]. This could explain different effects of enhanced cholesterol levels on the inhibitory action of ethanol in various signaling pathways. For example, the observed decrease in the inhibitory effect of ethanol on calcium or ROS responses in cells with enhanced cholesterol levels could reflect the reduced penetration of ethanol through lipid bilayers with increased rigidity caused by cholesterol [63]. On the other hand, ethanol could act directly as an inhibitor of some enzymes involved at later stages of degranulation; this could be an explanation of our finding that degranulation was not protected from the inhibitory effect of ethanol in cells pretreated with sMβ. In this context, Lisboa et al showed that phosphatidic acid produced by phospholipase D (PLD) plays an important role in promoting IgE-dependent signaling events within lipid microdomains in mast cells [64]. As a tool they used 1-butanol, which subverts production of phosphatidic acid to the biologically inert phosphatidyl butanol. Similarly, the presence of ethanol could lead to production of phosphatidyl ethanol instead of phosphatidic acid [65] and in this way inhibit FcεRI signaling. However, our and others’ data suggest that ethanol inhibits FcεRI signaling by another mechanism. We found that the inhibitory effect of ethanol on calcium response and ROS production was blocked by exposure of the cells to sMβ, which is unlikely to change production of PA and/or activity of PLD. Furthermore, it has been recently found that mice deficient in PLD1 and PLD2, which do not produce any PA, show no changes in early signaling events after FcεRI triggering [66].

**Fig 11:**
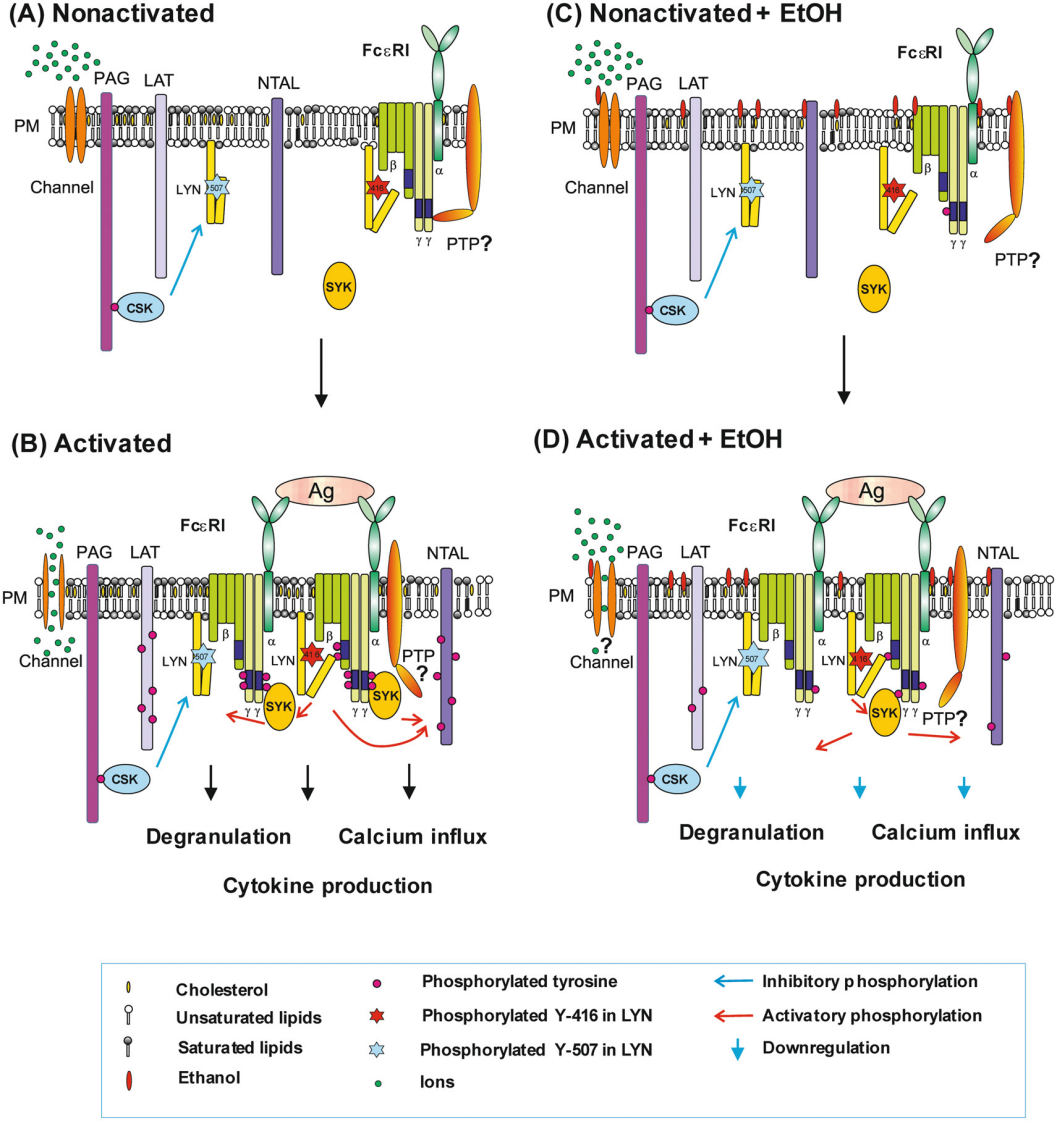
Model of FcεRI-mediated activation in ethanol-pretreated mast cells. In nonactivated cells (A), the topography of FcεRI and other signaling molecules, such as SRC family kinase LYN, protein tyrosine phosphatase (PTP), and adaptor proteins (LAT, PAG, and NTAL), prevents signaling. An important role in this process is played by the plasma membrane cholesterol. Aggregation of the FcεRI-IgE complexes by multivalent antigen (B) induces topographical changes that lead to formation of the FcεRI signalosome and enhanced tyrosine phosphorylation of the FcεRI β and γ subunits by LYN and SYK kinases. This results in enhanced degranulation, calcium response, cytokine production and numerous other events. In the cells exposed to ethanol and/or with reduced amount of cholesterol (C), the topography of plasma membrane molecules is slightly modified, resulting in increased tyrosine phosphorylation of some signaling molecules even in nonactivated cells. Aggregation of the receptor in ethanol-treated cells leads to suboptimal topographical changes resulting in reduced tyrosine phosphorylation of the FcεRI β and γ subunits by LYN and SYK kinases and/or enhanced activity of the corresponding phosphatases (D). This leads to reduced degranulation, calcium response, cytokine production and other events. Ethanol could also bind directly to some cytoplasmic or plasma membrane proteins, such as ion channel proteins, and in this way inhibit the cell signaling.

Model of ethanol action on mast cells and their activation by antigen is shown in Fig 11. Exposure of the cells to ethanol leads to changes in properties of plasma membrane and topography of plasma membrane components, resulting in the observed increase in basal tyrosine phosphorylation of some membrane proteins, including FcεRI. When IgE-sensitized and ethanol-treated cells are activated by antigen, tyrosine phosphorylation of the FcεRI β and γ subunits is reduced reflecting suboptimal topography and/or reduced activity of LYN and SYK kinases and/or enhanced activity of protein tyrosine phosphatases in the vicinity of the receptor. Ethanol could also bind to ion channels or some other plasma membrane or cytoplasmic proteins and in this way inhibit various signaling events, including degranulation, calcium response and production of cytokines.

The inhibitory effect of ethanol on mast cell activation was confirmed by experiments *in vivo* in which mast cells were sensitized locally by IgE, and ethanol and antigen was administered intraperitoneally and intravenously, respectively. The results of PCA assays, which reflect the activity of mast cells [67] support previous findings indicating that excessive ethanol consumption is associated with increased risk of infection [68]. Experimental studies with animal and human subjects given ethanol in a controlled setting showed suppression of the innate immunity and inflammation [69–71]. Attention was focused mainly on the inhibitory effect of ethanol on activation of macrophages [72,73], monocytes [74] and T cells [20,75–77]. Although previous studies also indicated that mast cells could be the target of ethanol [24,25], this is the first study showing that ethanol inhibits the earliest events in FcεRI signaling and demonstrate the inhibitory effect of ethanol on mast cells *in vivo*.

## Conclusions

In this study we found that 15 min treatment with non-toxic concentrations of ethanol *in vitro* inhibited antigen-induced tyrosine phosphorylation of the FcεRI β and γ subunits, SYK kinase, NTAL adaptor protein, PLCγ, calcium response, production of ROS, degranulation, and expression of cytokine genes in a dose-dependent manner. Early activation events were also inhibited by Mβ, suggesting that cholesterol could be involved. The role of cholesterol in the inhibitory effect of ethanol was supported by the finding that sMβ reduced the inhibitory effect of ethanol on calcium and ROS responses. The data support the lipid-centric theory of ethanol action on the initial stages of FcεRI signaling. The inhibitory effect of ethanol on mast cell activation was also observed in a mouse PCA model *in vivo*, explaining previous findings of the reduced inflammatory response associated with enhanced consumption of ethanol.
